# Successful treatment of multiple bilateral impactions - a case report

**DOI:** 10.1186/s13005-016-0122-0

**Published:** 2016-07-25

**Authors:** Michael Schubert, Peter Proff, Christian Kirschneck

**Affiliations:** 1 Private Practice, Albertstraße 5, 93047 Regensburg, Germany; 2 Department of Orthodontics, University Medical Centre of Regensburg, Franz-Josef-Strauss-Allee 11, 93053 Regensburg, Germany; 3 Department of Orthodontics, University Medical Centre of Regensburg, Franz-Josef-Strauss-Allee 11, 93053 Regensburg, Germany

**Keywords:** Case report, Diagnosis and treatment planning, Impaction, Guided eruption

## Abstract

**Background:**

Successful treatment of patients with multiple bilateral impactions can be an orthodontic challenge, but few reports on treatment planning and execution exist.

**Case presentation:**

In this case report, we describe the successful orthodontic treatment of a 16.3-year old female patient without systemic or genetic disease with initially nine persisting deciduous and nine impacted permanent teeth with complete root formation and closed apices in both jaws. After extraction of the deciduous and surgical exposure of the impacted permanent teeth, the Easy-Way-Coil™ system was used in conjunction with a skeletal anchorage (maxilla, BENEfit™ system) to guide the eruption of all impacted teeth. After a total treatment time of only 22.8 months all impacted teeth could be aligned successfully and a stable and functional class I occlusion was achieved. In addition, there were no adverse treatment effects such as anchorage loss, root resorptions or periodontal problems and an esthetic result could be achieved.

**Conclusions:**

The presented treatment approach thus proved to be highly effective in cases with multiple bilateral impactions with minimal side effects and considerably reduced treatment time.

## Background

A successful treatment of cases with multiple impactions poses a major challenge for most orthodontists in clinical practice and requires a meticulous treatment planning, preferably in collaboration with a dental surgeon. Apart from complicating the treatment and its predictability [1], treatment times usually increase significantly [2–4] and there is considerable concern that the impacted teeth could show or develop signs of invasive cervical root resorption (ICRR) or ankylosis [5], leaving the patient in the long term with a distinct hypodontia, if an extraction of these teeth becomes necessary.

Following the impaction probability of wisdom teeth, upper canines are the most likely teeth to be impacted, with a chance if about 1.59 % in the general populace, followed by lower second premolars, upper central incisors and the lower first premolar [6]. By contrast, impacted mandibular canines have a chance for impaction of only 0.35 % [7]. Since the occurrence of impacted maxillary canines is quite frequent, many studies have described successful treatment plans for aligning these teeth, in most cases involving a combined surgical-orthodontic approach [8–13]. However, due to the sporadic occurrence of additional impacted teeth in non-syndromal patients (about 0.32 % [6]), only a few studies report problems and treatment strategies [1, 7].

The Easy-Way-Coil™ (EWC) system used for traction in this case (Adenta, Gilching, Germany) has already been shown to be effective in aligning impacted upper canines [2, 14], but its efficacy for successful alignment of multiple bilaterally impacted teeth has not been investigated before. Compared to alternative appliances [10, 15, 16] and previous treatment approaches in cases with multiple impactions [1, 7], it enables a rotational control and variable direction of traction at a well-defined force-level, thus enabling a more controlled, comfortable and faster tooth alignment [14]. In addition, this system does not rely on elastics for force generation, which apart from often exerting undefined force levels have to be replaced quite frequently, or on lever designs, which apart from reduced patient comfort and hygienic problems are prone to be damaged easily [14].

The female patient described in this report was 16.3 years old at the beginning of treatment and had initially nine persisting deciduous teeth, whereas nine permanent teeth with complete root formation and closed apices were found to be impacted.

## Case presentation

### Diagnosis and etiology

The patient, a 16.3 year-old girl, was in excellent health, both physically and emotionally, with no known adverse drug reactions or allergies and good oral hygiene. The major concern of both the patient and the referring general dentist was the persistence of several deciduous teeth. Possible systemic, endocrine, metabolic or genetic-syndromal disorders as possible etiologic factors could not be detected by a physician and endocrinologist, to which the patient was referred prior to orthodontic examination. In addition, there was no family history of eruption failure of permanent and persistence of deciduous teeth. The first deciduous upper molar on the right side (tooth 54) was extracted 1 year earlier by the referring general dentist, but no spontaneous eruption of the permanent first premolar could be achieved within this timeframe (Table 1). Thus and due to no previous referral by the general dentist or presentation of the patient for orthodontic planning, treatment was started quite late.

**Table 1:** Timeline of patient diagnosis and treatment

1.	Presumed retention of tooth 14 with extraction of deciduous tooth 54 by the referring dentist to facilitate tooth eruption and radiological imaging (OPG)
2.	1 year later: Initial orthodontic screening and referral to physician and endocrinologist
3.	Diagnostic orthodontic records: Clinical examination and model analysis
4.	Beginning of treatment within the maxilla •placement of fixed orthodontic appliance and placement of the skeletal anchorage •right upper quadrant: extraction of persisting deciduous teeth, surgical exposure, bonding of EWC® appliances and repositioning of mucoperiosteal flap
5.	1 week later: First activation of EWC® springs within right upper quadrant
6.	4 weeks later: extraction of persisting deciduous teeth within the left upper quadrant, surgical exposure, bonding of EWC® appliances and repositioning of mucoperiosteal flap
7.	1 week later: First activation of EWC® springs within left upper quadrant
8.	about 3 months post treatment start within upper jaw: treatment start within the mandible •extraction of persisting deciduous teeth and spontaneous eruption of premolars •placement of fixed orthodontic appliance •right lower quadrant: surgical exposure of tooth 43, bonding of EWC® appliance and repositioning of mucoperiosteal flap
9.	1 week later: First activation of EWC® spring within right lower quadrant
10.	Every 4 weeks after respective first activation (upper/lower jaw): Clipping of the EWC® coil spring (activation) by 2 (1) mm
11.	At tooth eruption: removal of coil spring and further traction by a Powertube™25 attached to lingually, then - when possible - buccally bonded buttons and the circumferential 0.017” × 0.025” SS arch-bow
12.	Superelastic NiTi circumferential arch-bow (final extrusion and levelling) with correction of lower midline deviation by canine uprighting (superimposed power chain over arch-bow from lateral right incisor to left first premolar)
13.	Subsequent TMA circumferential arch-bows (finishing)
14.	Removal of skeletal anchorage

**Fig. 1:**
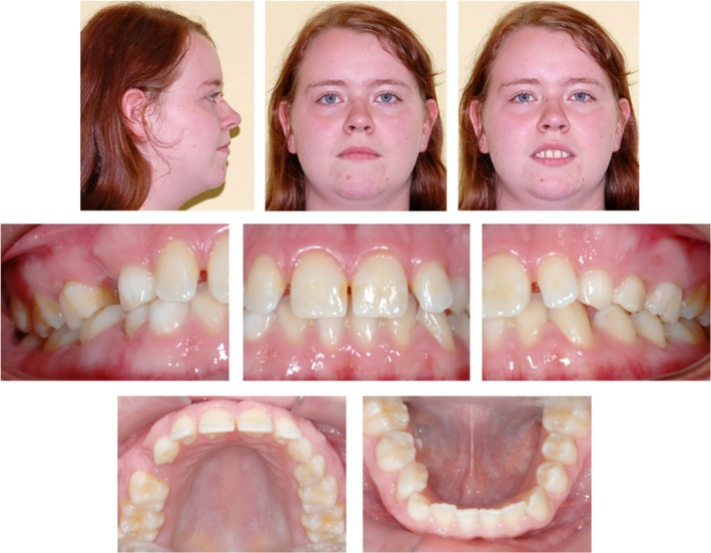
Pretreatment extraoral and intraoral photographs

**Fig. 2:**
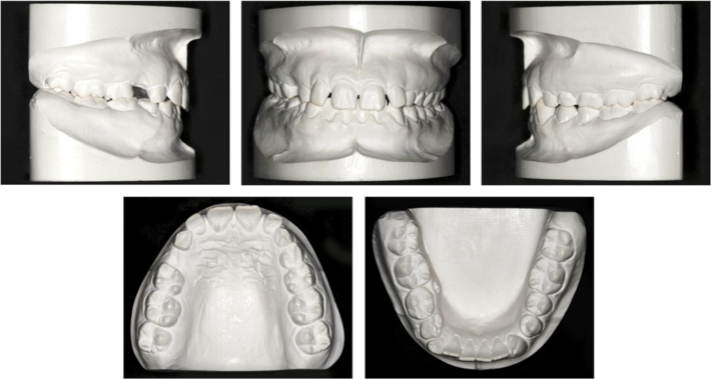
Pretreatment orthodontic study models

**Fig. 3:**
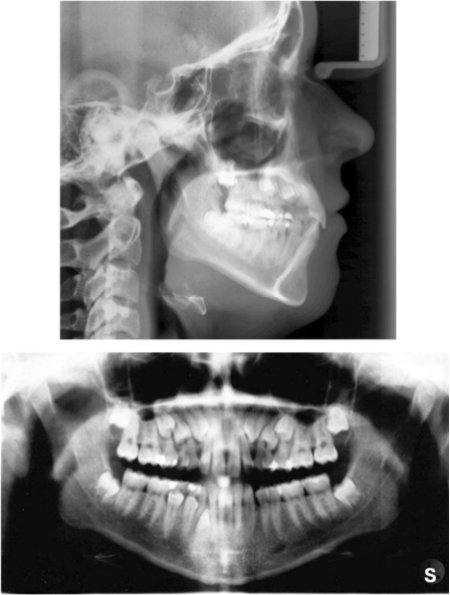
Pretreatment lateral cephalometric and panoramic radiographs

The clinical examination and study model analysis (Figs. 1 and 2) in conjunction with the panoramic radiograph (Fig. 3), taken by the referring general dentist, showed initially nine persisting deciduous teeth – all deciduous canines and molars of the upper jaw and those on the right side of the mandible, minus the already extracted upper right deciduous molar. All upper permanent canines and premolars were impacted and malpositioned. The lower right canine, which was severely rotated, was also impacted to a higher and the lower right first and second premolars to a lesser degree, whereas the corresponding teeth on the lower left side were in good occlusion and showed no signs of impaction. No adequate root resorption of the deciduous teeth could be detected radiologically with exception of the lower deciduous molars on the right side. Radiographically the impacted teeth showed no evident signs of ankylosis. Some physiological spacing of the incisors was present and both the first permanent molars as well as the deciduous canines were in good occlusion and had a class I relationship with a shallow curve of Spee in both jaws. The second permanent molars were also in occlusion and all four third permanent molars present radiologically within their germ stage. No reduction of the extraction space due to a mesial or distal drifting of the neighboring teeth was evident. The patient also showed a lower alveolar midline deviation to the right side (about 2 mm), most likely due to the unilateral failure of eruption at the lower right side, which caused a more mesial eruption and inclination of the lower left permanent canine and the lower incisors, as evidenced by the gap formed between the canine and lower left premolar.

3D CBCT diagnostic imaging was declined by the patient and her guardian due to concerns about a potential health risk by the radiation exposure. The cephalometric analysis according to Ricketts (Table 2, Fig. 3) revealed a vertical dolichocephalic growth type, hyperdivergent jaws and a large mandibular plane angle, thus a further vertical development had to be avoided during treatment. In addition, the mandible was found to be prognathic with an ortho- to retrognathic upper jaw, resulting in a slight skeletal class III, which was dentally compensated (class I molar relation). Both approximately normally inclined upper and lower incisors were distinctly protruded, whereas Ricketts’ E-line indicated a retrusion of the upper lip due to the proganthic lower jaw. Furthermore, overjet and overbite were increased.

**Table 2:** Cephalometric measurements

Ricketts analysis	Pretreatment (16 years, 4 months)			Posttreatment (19 years, 8 months)		
	Norm	Value	Deviation	Norm	Value	Deviation
Cranial relations						
Facial axis (°)	90 ± 3.5	88.4	vertical growth type	90 ± 3.5	89.0	dolichofacial face type
Mandibular plane (°)	23.6 ± 4	34.0		29.6 ± 4	29.9	
Mandibular arc (°)	26 ± 4	30.1		27.7 ± 4	26.4	
Lower facial height (°)	45 ± 4	54.1		45 ± 4	58.1	
Maxillary depth (°)	90 ± 3	87.4	maxilla ortho- to retrognathic	90 ± 3	89.7	maxilla orthognathic
SN-Palatal plane (°)	7.3 ± 3	−3.3	anterior rotation of maxilla	7.3 ± 3	−1.2	less anterior rotation of maxilla
Facial (angle) depth (°)	89.4 ± 3	84.6	mandible prognathic	90.6 ± 3	85.7	mandible prognathic
Facial taper	68 ± 3	61.4	posterior rotation of mandible	68 ± 3	64.4	less posterior rotation of mandible
Convexity of point A (mm)	−0.4 ± 2	2.6	slight skeletal class III	−1.6 ± 2	3.8	skeletal class III
Denture Relations						
Upper incisor to A-Pog (mm)	3.5 ± 2	10.7	distinctly protruded	3.5 ± 2	9.6	slightly less protruded
Upper incisor to FH (°)	111 ± 4	105.5	slightly retroinclined	111 ± 4	107.4	normally inclined
Upper molar to PtV(mm)	19.3 ± 1	15.2	upper dental arch retruded	22.7 ± 1	18.3	upper dental arch retruded
Lower incisor to A-Pog (mm)	1 ± 2	6.5	distinctly protruded	1 ± 2	7.1	distinctly protruded
Lower incisor inclination (°)	22 ± 4	24.5	normally inclined	22 ± 4	23.4	normally inclined
Interincisal angle (°)	130 ± 6	132.4	normal	130 ± 6	131.8	normal
Molar relation (mm)	−3.0 ± 2	−2.6	Angle class I	−3.0 ± 2	−1.1	Angle class I
Incisor Overjet (mm)	2.5 ± 1	4.6	increased	2.5 ± 1	2.5	normal
Incisor Overbite (mm)	2.5 ± 1	3.2	increased	2.5 ± 1	2.2	normal
Esthetic Relations						
Lower lip – E-Plane(mm)	−2 ± 2	−3.9	lower lip slightly retruded	< -2 ± 2	−4.3	lower lip slightly retruded

The radiologically evident complete root development of all permanent impacted teeth with closed apices in conjunction with the persisting deciduous teeth without distinct signs of root resorption in absence of genetic-systemic causes formed the basis for our diagnosis of idiopathic multiple bilateral impactions, confirmed by the failure of spontaneous eruption of the first upper left premolar after extraction of the corresponding deciduous predecessor.

### Treatment objectives

The main treatment objective was to align the nine impacted teeth at the occlusal level by means of controlled guided traction after their surgical exposition and ligation and after extraction of the preceding deciduous teeth. Secondary objectives were a levelling of the arches, a correction of the lower dental midline shift, establishing a physiological overjet and overbite and achieving a stable and functional occlusion. We opted for treatment with a fixed multi-bracket appliance in conjunction with the Easy-Way-Coil System™ (EWC) [14]. To minimize treatment time, a simultaneous alignment of all impacted upper six permanent teeth was planned (4 weeks discrepancy in start of treatment between right/left side). Vertical and horizontal relations as well as dental class I were to be kept stable during treatment and the risk of root resorption at the second incisors was minimized by excluding them from the appliance. Due to the higher treatment complexity of the maxilla, treatment within the mandible was started at a later date.

The patient and her legal guardians were informed and motivated about the necessity of good oral hygiene and compliance during the treatment as well as about the expected duration of treatment and the risks involved - particularly root resorptions and failure of eruption, which would necessitate a later implant and restorative treatment. Informed consent was obtained and ethical regulations according to the Declaration of Helsinki (1964) and its later amendments were observed at all times.

### Treatment alternatives

The best choice of treatment was discussed and considered both with the patient and an oral surgeon and a combined surgical-orthodontic approach with simultaneous traction of the impacted teeth by means of an Easy-Way-Coil™ was deemed to be most promising and time-efficient.

The most simple approach to treat multiple impactions would have been to just extract the persisting deciduous teeth and wait for a spontaneous eruption. However, the advanced age of the patient, the complete root development and the already failed attempt by the general dentist made a success of this approach seem unlikely.

An alternative method described by Schmidt and Kokich [17] to extract the deciduous teeth and only expose the impacted teeth surgically without traction to facilitate spontaneous eruption, which for canines usually occurs after 6–8 months [7], was rejected by the patient for cosmetic reasons. This also excluded the possibility of a surgical luxation of the impacted teeth to facilitate tooth movement. In addition, the impactions were considered to be too deep for an exposure without traction to be successful. For this reason, we also opted for a closed instead of an open eruption technique in this case [9, 18].

If the impacted teeth were ankylosed, an extraction and consecutively implant-restorative treatment would be indicated. This option was considered as potential alternative, if the primary therapy failed in case of confirmed or developing ankylosis or invasive cervical root resorption during treatment, whereas at baseline no ankylosis of the impacted teeth could be confirmed. In this case a mesialisation of the upper permanent molars by means of skeletal anchorage and a mesial sliding appliance [19] could have been used to reduce the resulting gaps, enable an occlusal alignment of the third molars and reduce the number and extent of necessary implants and restorative treatment.

A sequential extraction of persisting deciduous teeth [7] and also sequential traction of the impacted teeth individually as described by Krey et al. [20] could have reduced the risk of extensive tooth loss in case of alignment failure. This approach, however, was rejected due to the unwillingness of the patient to accept prolonged treatment times, her already advanced age for treatment and a persisting deciduous dentition. The patient rather preferred implant-restorative treatment in case of treatment failure.

### Treatment progress

**Fig. 4:**
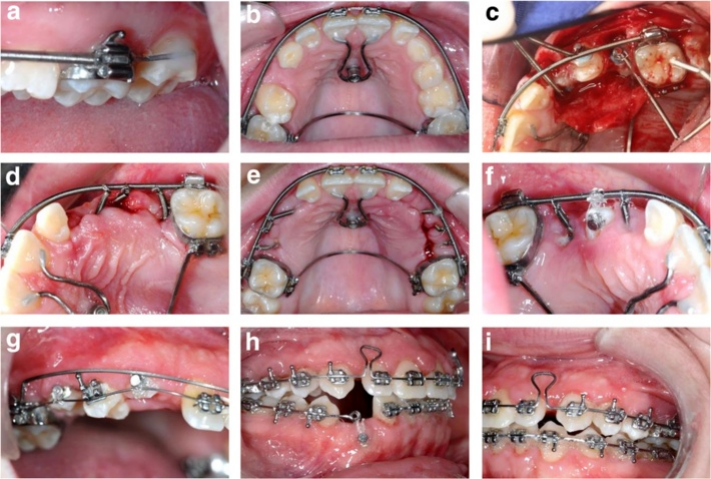
Progress photographs during treatment. For details regarding the treatment progress as depicted in panels **a**-**i**, please refer to section treatment progress

At the beginning of treatment brackets (slot 0.018”) were bonded only to the upper central incisors and bands were placed on the first molars. These were connected by a transpalatinal arch (Burstone, 0.032” × 0.032” stainless-steel) for anchorage. Second molars were stabilized by connecting them with the first molars with a passively bonded partial arch-bow (0.017” × 0.025” stainless-steel) (Fig. 4a).

At the level of the second transversal palatal ridges one mini-screw of the BENEfit™ system (2.0 × 11 mm, dental line, Birkenfeld, Germany) [21] was inserted into the palate to carry a screwed standard abutment with a welded 1.1 mm stainless-steel wire. This wire was shortened, bent to the upper central incisors in shape of a U and bonded to their palatal surface (Fig. 4b). The upper central incisors and first molars were then connected with a 0.017” × 0.025” stainless-steel arch-wire (straight-wire technique) within a passive closed coil spring (Fig. 4b). The lateral incisors were intentionally left out of the fixed appliance to avoid any root movement and contact with the impacted canines.

Treatment was started at the upper right quadrant. After extraction of the persisting deciduous teeth a mucoperiosteal flap was raised and the impacted permanent teeth were surgically exposed up to the assumed cemento-enamel junction. Three EWC™ appliances were bonded to the individual teeth at the palatal surface (canine) and at the buccal surfaces (premolars) and directed towards the arch-bow used for anchorage (Fig. 4c). The mucoperiosteal flap was then repositioned and fixated with surgical sutures (closed eruption technique).

After 1 week the sutures were removed and the EWC™ springs activated. The direction of traction was chosen to be disto-buccal for the canines and buccal for the premolars. A suitable anchorage spot was chosen on the circular arch-bow and a gap introduced into the passive closed coil spring by gently closing a ligature cutter at this location. This way a displacement of the ligature wire used for connecting the EWC™ spring with the arch-bow was effectively prevented. The correctly aligned EWC™ springs were then shortened to create a gap of exactly 2 mm (canine) and 1 mm (premolars) between arch-bow and the spring. By gently closing the ligature cutter 1 mm proximal to the shortened end of the EWC™ spring, an eyelet consisting of the terminal three coils was formed, which was connected seamlessly to the gap formed at the arch-bow coil spring by means of a ligature wire (Fig. 4d). The EWC™ spring activation of 2 and 1 mm ensured a predictable force level of 0.32 N and 0.16 N, respectively.

**Fig. 5:**
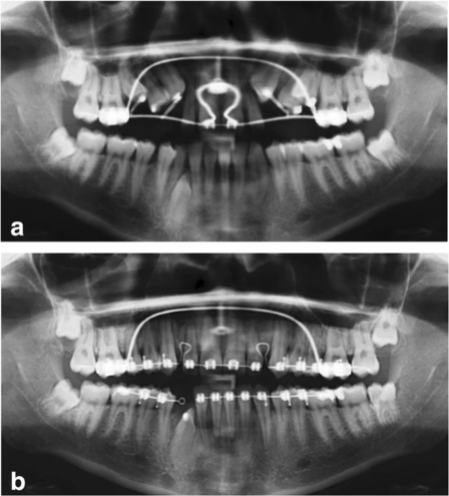
Sequence of panoramical radiographs during the orthodontic treatment. **a** Traction of impacted *upper* teeth; **b** Space closure and traction of *lower right* canine

Four weeks later the same procedure was performed at the contralateral upper left jaw side (Fig. 5a). At the same time the EWC™ springs at the right side were reactivated by clipping 2 mm (canine) and 1 mm (premolars) off the springs at their anchorage point and re-fixating them with a ligature wire (Fig. 4e). This procedure was repeated every 4 weeks at both upper jaw sides until the impacted teeth erupted. Then the EWC™ springs were removed and lingual buttons attached to the occlusal surfaces of the premolars (Fig. 4f) and buccal surfaces of the canines (Fig. 4g). The teeth were then further moved in buccal direction by means of a PowerTube™25 (Ormco B.V., Netherlands) until brackets could be bonded to the labial surfaces (Fig. 4g). Final extrusion and levelling of all impacted teeth was achieved by a superelastic Nickel-Titanium (NiTi) arch-wire initially (Fig. 4g) and subsequent rigid TMA (Titan-Molybdenum-Alloy) arch wires.

After the deciduous teeth had been extracted in the lower jaw, the permanent premolars at the lower right side erupted spontaneously (Fig. 5a). A fixed multi-bracket appliance was bonded in preparation for aligning the impacted lower right canine, which was performed in analogy to the technique used in the upper jaw (surgical exposure, 0.017” × 0.025” stainless steel anchorage arch-bow with passive closed coil spring, EWC™ system and 2 mm activation). To prevent intrusion and tipping of the incisors at the lower right side, the continuous arch-wire was separated and only the lateral tooth segment used for anchorage (Figs. 4h and 5b).

After removing the upper skeletal anchorage, remaining spaces in the upper dental arch were closed and incisors slightly re- and intruded with a T-loop bow (0.017” × 0.025” TMA, Fig. 4h/i) to achieve physiological overjet and overbite. The lower alveolar midline deviation was corrected during levelling by uprighting the mesially inclined lower right canine into the gap to the first premolar with consequent distalisation of incisors aided by a buccal power chain placed on top of the arch-bow from the lower right second incisor to the left first premolar (Fig. 4i).

### Treatment results

Total treatment time was 22.8 months. The required time for aligning the upper jaw from surgical exposure to finishing with a 0.017” × 0.025” TMA arch-bow was 19.6 months with a total 26 sessions in the office. The corresponding treatment time for the lower jaw was 20 months with a total of 19 office visits.

**Fig. 6:**
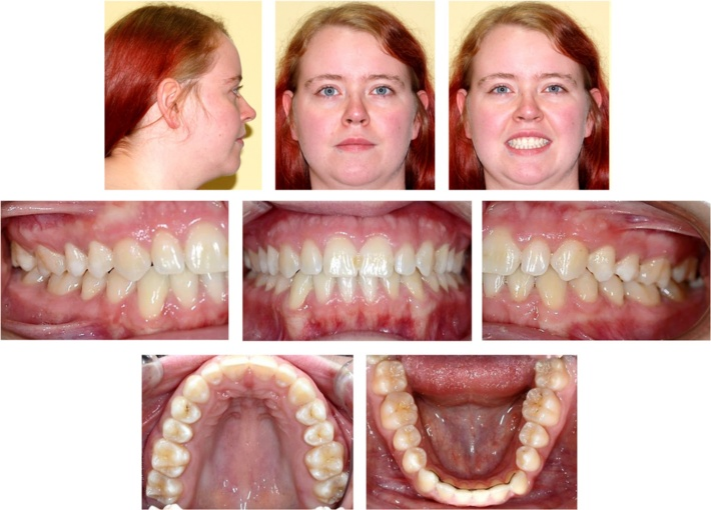
Posttreatment extraoral and intraoral photographs

**Fig. 7:**
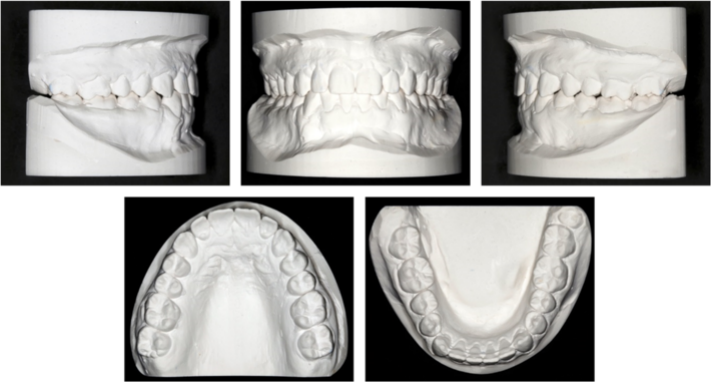
Posttreatment study models

**Fig. 8:**
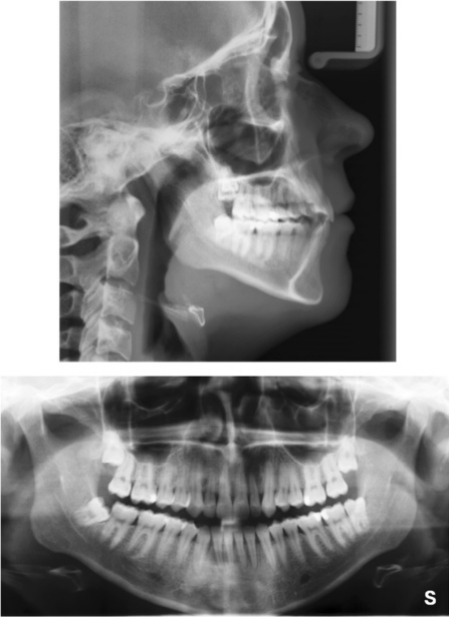
Posttreatment lateral cephalometric and panoramic radiographs

**Fig. 9:**
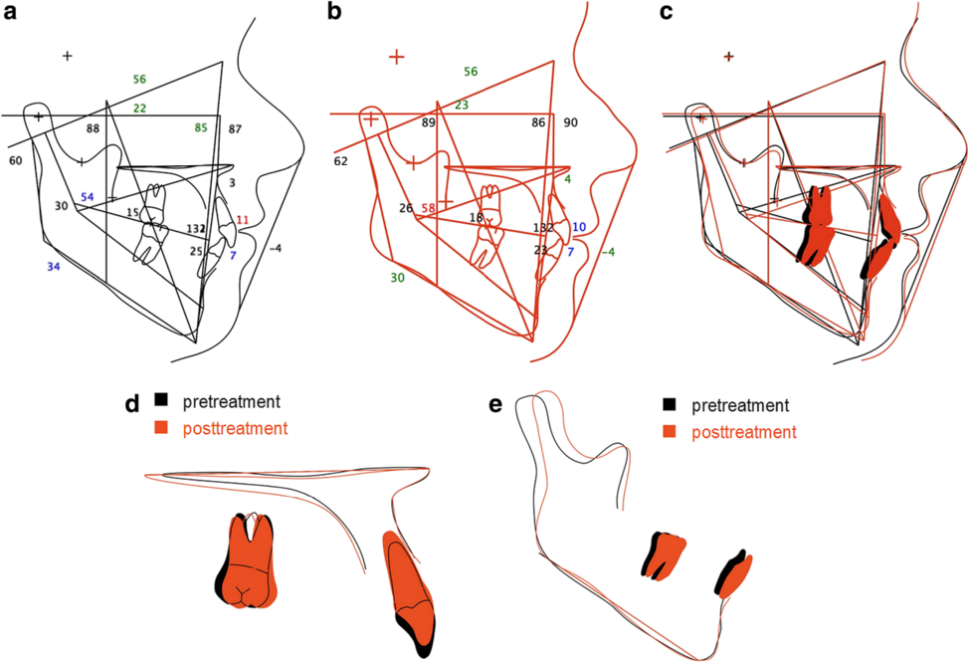
Cephalometric tracing and superimposition. **a** Pretreatment cephalometric tracing; **b** Posttreatment cephalometric tracing. The numbers represent the angular and metric measurements in degree/mm. **c** Superimposition on the sella-nasion plane at sella; **d** Superimposition of the maxilla on the maxillary spinal plane (NL) at the anterior nasal spine; **e** Superimposition of the mandible on the mandibular plane (ML) at menton

All impacted teeth could be successfully aligned within the levelled dental arches. Class I canine and molar relation were maintained at both sides (Figs. 6 and 7) and the patient (19.7 years) was happy with the treatment result. The cephalometric analysis after the end of treatment (Table 2, Fig. 8) as well as the superimposition of pre- and posttreatment tracings (Fig. 9) showed a slight intensification of the mesial skeletal tendency to a skeletal class III jaw relation (dentally compensated), caused by anterior rotation of the mandible, whereas the vertical hyperdivergence of the upper and lower jaw decreased by posterior rotation of the maxilla and anterior rotation of the mandible. The protrusion of the upper, but not lower incisors, both normally inclined, was reduced during treatment. Upper incisors were intruded. The lower alveolar midline deviation could be successfully corrected and a physiological overjet of 2.5 mm and overbite of 2.2 mm was achieved. The retrusion of the upper lip remained unchanged during treatment due to the still prognathic mandible.

For retention purposes, the patient was instructed to wear a removable Hawley-retainer for 24 h a day within the first 6 months and then to gradually reduce wear time (only at night, every second night etc.). In addition, a coaxial annealed retainer wire (Penta-One™ 0.0215”, Masel Orthodontics, Carlsbad, USA) was bonded to the palatinal surfaces of the lower incisors and canines due to the severe rotation of the aligned lower right canine and thus increased risk of rotational relapse [22].

A total of nine deciduous teeth were extracted and seven permanent teeth surgically exposed, ligated and aligned. The two initially impacted permanent premolars at the lower right side erupted spontaneously after extraction of the deciduous molars and did not require additional traction. Post-treatment clinical examinations and radiographs (Fig. 8) showed no indications of avital or traumatized teeth, root resorption, periodontal problems (probing depths < 3 mm for all teeth and locations) or other iatrogenic damages. At the end of treatment the patient was referred to the oral surgeon for possible extraction of the third molars, since proper eruption of the lower right molar seemed unlikely due to the radiographically evident lack of space and problematic angulation.

## Discussion

When aligning impacted teeth proper anchorage is of utmost importance, if undesired side effects, such as intrusion or tipping of adjacent teeth, are to be avoided. If sufficient dental anchorage cannot be achieved, skeletal anchorage techniques should be used. In general, these can be classified in two categories: osseointegrated dental implants including central palate implants and non-osseointegrated miniplates and mini-screws.

The stability of osseointegrated implants is very high and their positioning can be optimized – away from the dental roots – to avoid damaging the dentition. Both advantages, however, do not outweigh the primary disadvantage of repeated surgery (insertion and removal), thus we used non-osseointegrated mini-screws for skeletal anchorage in this case. Due to the favorable bone quality in the anterior center of the palate [23], this location is used for skeletal anchorage by the BENEfit™ system [21]. To increase biomechanical stability the diameter of the mini-screws used in this system is higher than that of conventional mini-screws used within the alveolar process. Additional stability in this area is generated by the rigidity of the attached mucosa [15]. Both aspects increase the success rate of this system to 94.7 % in relation to its failure rate [21]. To prevent direct forces and moments on the singular mini-screw implant, we chose an indirect mode of anchorage by redirecting the reactive forces via the arch-wire and the upper central incisors to the mini-screw.

Daskalogiannakis and McLachian [24] could show that mean tooth movement velocity is twice as high, if forces are applied continuously, compared to a discontinuous force application. However Ballard [25] found an increased incidence of external root resorption during continuous force application. Thus as a compromise, we used the EWC™ system in this case for traction of the impacted teeth, which applied a continuous decreasing force for a 4-week-interval, whereas total force delivery was discontinuous, since the stainless steel spring needed to be reactivated every 4 weeks.

Recommendations for the optimal force level for the traction of impacted teeth differ considerably, when reviewing the literature. The inhomogeneity of the patient collectives and techniques used for aligning impacted teeth makes meta-analyses and a systematic evidence-based evaluation difficult. One common factor, however, can be inferred: traction should be performed with low instead of high forces. In our case we followed the recommendations of Adrian Becker [26], using a force level of 0.2 N to 0.4 N/cm^2^ of dental root surface in direction of movement. Different movement directions thus require different force levels, which could be assessed by the root rating scale of Ricketts et al. [27]. A disto-buccal traction of the canine was calculated to require a force level of 0.15-0.3 N, based on a reactive root surface of 0.75 cm^2^. The lateral extrusive movement of premolars, which have a root rating scale of 0.5 cm^2^ each, needed a force level between 0.1 N and 0.2 N. These different force levels could be achieved by a 1 mm or 2 mm activation (shortening) of the EWC™ spring, which yielded a predictable and reproducible mean force level of 0.16 N and 0.32 N, respectively, without the need for corroboration by a spring scale.

Apart from a defined and controlled force application, the major advantage of the EWC™ system used in this study is the achieved rotational control compared to many other traction systems. The traction of impacted canines usually generates a distinct rotational moment, since forces are applied eccentrically due to the location of the attachment at the palatinal surface of the impacted tooth [28]. Based on the applied force level of 0.3 N and a mean distance of the attachment from the tooth axis of 2-3 mm, a rotational moment of 0.6–0.9 Nmm is generated, which would in turn lead to an undesired rotation of the impacted tooth during alignment. This would require a correction later on, which not only takes additional time, but also poses considerable risk for rotational relapse, one of the most frequent forms of relapse [22]. It has been shown that the rigidity of the EWC™ spring can effectively produce an anti-rotational moment of 0.75 Nmm, which reduces or even negates the rotational moment during traction [14]. Due to the reduced risk of relapse, palatinal retainer wires are usually not required [14].

The treatment of the impacted lower right canine was started 10 months later than treatment of the upper jaw due to the supposedly lower complexity and time demand. This decision, however, proved to be unfortunate, since alignment of the impacted upper teeth proceeded far more quickly than anticipated compared to the vertical development of the lower canine. This miscalculation was due to the fact that there are several reports in the literature about treatment times for aligning impacted upper canines [2, 3], but none for lower canines. The longer treatment time for aligning the lower canine was probably due to differences in bone structure, with more cortical bone present in the mandible. Total treatment could have been completed sooner, if the surgical exposure and traction of the impacted lower canine had been started earlier. In addition, the lower right canine was distinctly rotated and most likely situated with its lateral root area within compact bone due to its oval root shape. In this respect, additional studies about the most favorable treatment timing, especially for lower canines, would be helpful.

Few studies reported about the alignment of multiple bilateral impactions of permanent teeth. One of these is a case report by Zuccati and Doldo [7], published in 2010. The initial situation of their case was quite similar; however, the treatment differed considerably. In our case we used a skeletal anchorage to simultaneously align six impacted upper permanent teeth, whereas Zuccati and Doldo used remaining deciduous molars for dental anchorage while sequentially aligning the impacted teeth. Thus their treatment took 3.5 years and a total of 88 visits by the patient, whereas the approach presented in this case report could effectively reduce treatment time to 1.9 years. This discrepancy is due not only to their sequential treatment approach, but also to the elastic ligatures used for traction, which had to be changed every 2 weeks. In comparison, the stainless steel EWC™ spring used for traction in our case only needed reactivation every 4 weeks. An additional time-reducing factor was the skeletal anchorage implemented in our case [29], enabling a simultaneous alignment of all impacted teeth without side effects to the adjacent teeth as seen in dental anchorage.

Another important factor in orthodontic treatment of cases with impactions, which is however considered only by few authors [8], is the personal view of the patient concerning the surgical and orthodontic procedures. The EWC™ system used in this study facilitated oral hygiene and reduced discomfort due to the closed eruption technique. In addition, due to its rigidity and simplicity, it is unlikely to be deformed or damaged by the patient and rather comfortable to wear in contrast to other treatment tools such as cantilever mechanics. In addition, the directed buccal eruption of the impacted teeth obviated the need for an occlusal bite block, otherwise necessary for correcting the crossbite situation of the canine, and did not impede masticatory function of the patient. Patient compliance and treatment tolerance was therefore quite high during the treatment process, most likely due to increased patient comfort.

## Conclusions

- The presented combined surgical-orthodontic treatment procedure with the EWC™ traction system enabled an efficient and controlled uprighting and buccal eruption of the formerly palatinally impacted canines and the premolars with good rotational control during traction.
- By means of skeletal anchorage, a simultaneous alignment of the impacted upper teeth could be achieved, thus considerably reducing treatment time and necessary visits at the orthodontic office.

## Abbreviations

EWC, easy-way-coil™; ICRR, invasive cervical root resorption; NiTi, nickel-titanium; TMA, titan-molybdenum-alloy
